# Extensive unilateral nevus comedonicus with an inflammatory component^⋆^

**DOI:** 10.1016/j.abd.2021.01.012

**Published:** 2022-11-04

**Authors:** Gessica Ramos Barroso Diniz, Flávia Vasques Bittencourt

**Affiliations:** a Dermatology Service, Hospital das Clínicas, Universidade Federal de Minas Gerais, Belo Horizonte, MG, Brazil

Dear Editor,

This case report describes a four-year-old child, followed for three years, with normochromic plaques covered by a cluster of follicular openings filled with keratin, comedo-like, along a large extension of the right side: cervical and retroauricular regions, trunk, buttocks, lower limb and foot.

The lesions were located along the Blaschko's lines (Figure 1, Figure 2). Dermoscopy showed more clearly the agminated keratotic plugs (Fig. 3). The lesions were present at birth, and those in the cervical region were often the site of inflammation and secondary infection, requiring recurrent cycles of oral antibiotic therapy during follow-up and surgical removal of the inflamed portion. The child had age-appropriate neuropsychomotor development, with no complaints related to other systems and no family history of relevant diseases. There was no ocular, neurological or skeletal involvement.

**Figure 1:**
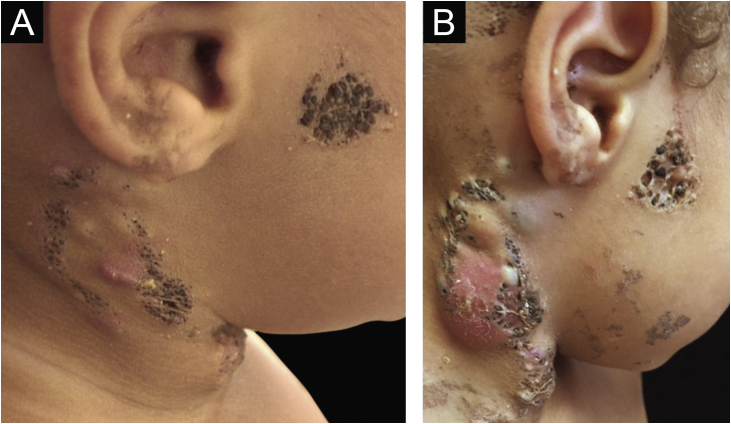
(A) Plaques covered by clusters of comedones, with a small erythematous area and formation of cysts on the right lateral cervical region. (B) Same lesion with worsening of the inflammatory condition.

**Figure 2:**
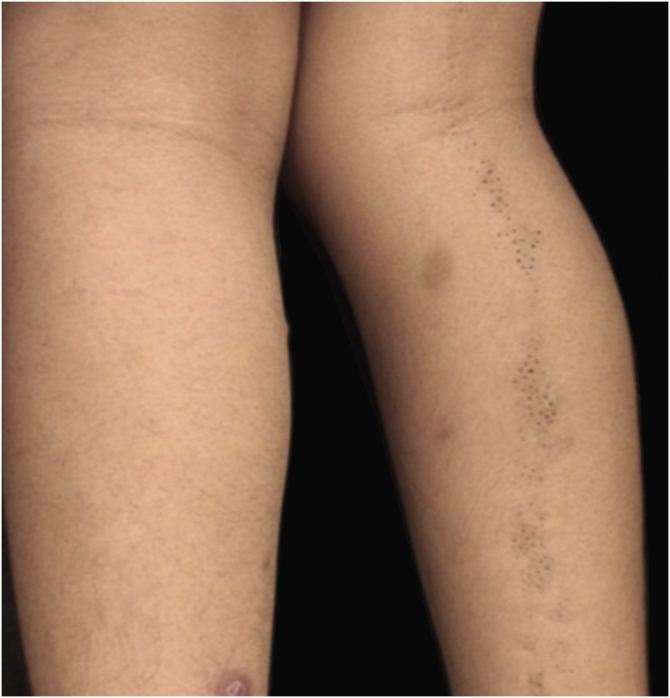
Linear lesion formed by a cluster of comedones following Blaschko's lines on the right lower limb.

**Figure 3:**
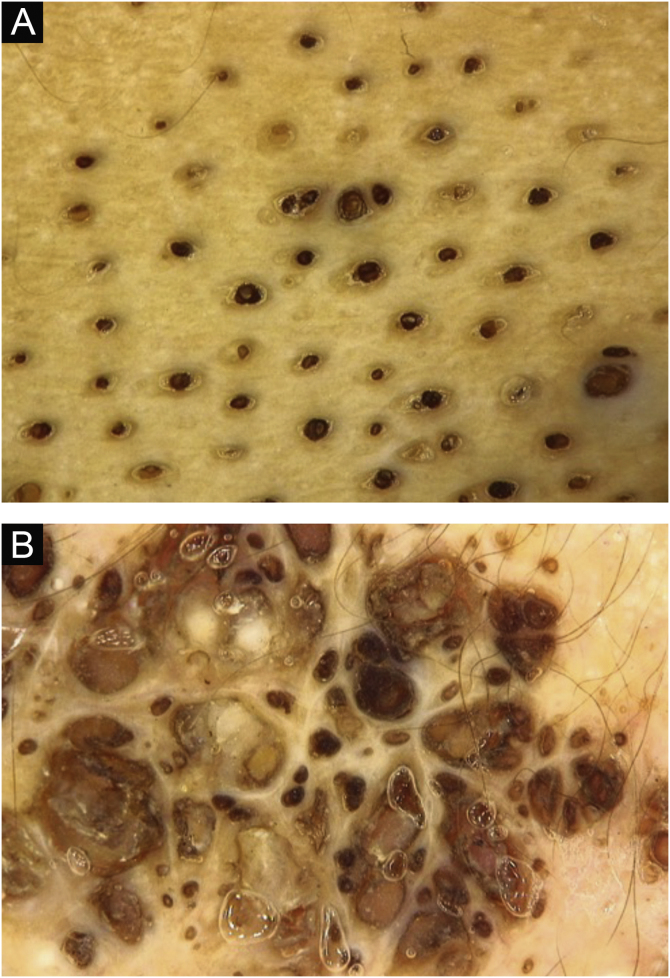
Dermoscopy: dilated follicular openings forming groups of keratinous plugs.

Nevus comedonicus is a rare type of epidermal nevus, characterized by a developmental change affecting the pilosebaceous unit. The most frequently affected regions are the face, cervical region, and trunk. It affects both sexes equally, can be congenital, and, in most cases, appears in children before the age of ten.^1^ It arouses interest due to the scarcity of cases reported in the literature and the diverse clinical presentations, whether as isolated lesions or extensive cases.

The first report of nevus comedonicus was presented in 1875 by Kofmann, and a little over 200 cases have been reported in the literature since then.^2^ Some authors divide nevi comedonicus into two groups: the non-inflammatory type, consisting of asymptomatic comedonal lesions, with purely aesthetic consequences, and the inflammatory one, which is rarer, more exuberant, with inflammatory cysts and recurrent infections, as the case described here.^3^

The diagnosis of nevus comedonicus is usually a clinical one and may be aided by dermoscopy, especially to differentiate it from other epidermal nevi, such as nevus sebaceous.^1^ Histopathology shows dilated and elongated follicular infundibula, with basophilic lamellar corneal content.

This rare hamartomatous condition may be associated with genetic syndromes affecting other systems, and it is important to investigate skeletal, ocular, and central nervous system alterations, which, if present, constitute the nevus comedonicus syndrome.^4^ Despite the extent of the lesions in the patient described in the present report, along the lines of Blaschko, which suggested a mosaic pattern, no associated systemic changes were identified.

The treatment can be topical with the use of emollients, corticosteroids in inflammatory lesions, and keratolytic agents. The use of topical tretinoin has been reported, but there is limited data regarding its efficacy.^4^ The use of oral isotretinoin has been shown to be ineffective in most patients; however, it may be considered an option in disseminated cases.^3^ There have also been some random reports on the use of laser treatment with partial response.^5^ Surgery is an excellent option in localized lesions. Although the lesions were extensive in the case described here, affecting the right side of the body, only those located on the cervical region showed inflammation and infection, which prompted surgical approach in this symptomatic and restricted area, with good response.

## Financial support

None declared.

## Authors' contributions

Gessica Ramos Barroso Diniz: Collection of data; drafting and editing of the manuscript or critical review of relevant intellectual content; approval of the final version to be submitted.

Flávia Vasques Bittencourt: drafting and editing of the manuscript and critical review of relevant intellectual content; approval of the final version to be submitted.

## Conflicts of interest

None declared.

## References

[1] Kamińska-Winciorek G., Spiewak R. (2013). Dermoscopy on nevus comedonicus: a case report and review of the literature. Postepy Dermatol Alergol..

[2] Kirtak N., Inaloz H.S., Karakok M., Erguven H.G., Ozgoztasi O. (2004). Extensive inflammatory nevus comedonicus involving half of the body. Int J Dermatol..

[3] Guldbakke K.K., Khachemoune A., Deng A., Sina B. (2007). Naevus comedonicus: a spectrum of body involvement. Clin Exp Dermatol..

[4] Ferrari B., Taliercio V., Restrepo P., Luna P., Abad M.E., Larralde M. (2015). Nevus comedonicus: a case series. Pediatr Dermatol..

[5] Caers S.J., Van der Geer S., Beverdam E.G., Krekels G.A., Ostertag J.U. (2008). Successful treatment of nevus comedonicus with the use of the Erbium Yag laser. J Eur Acad Dermatol Venereol..

